# Effectiveness of Zonal Observation in Reducing Restrictive Practices on a Male Psychiatric Intensive Care Unit (PICU) Over a One-Year Period

**DOI:** 10.1192/bjo.2025.10448

**Published:** 2025-06-20

**Authors:** Yetunde Arowora, Faizaan Syed, Shantala Satisha

**Affiliations:** Kent and Medway NHS Trust, Dartford, United Kingdom

## Abstract

**Aims:** We aimed to reduce the use of seclusion, 1:1 and 2:1 observations in our PICU, without compromising safety, by introducing zonal observation levels which is considered less intrusive, allowing greater privacy for the patient and better engagement.

Hypothesis: We expect a reduction in number of enhanced observations with no change in levels of aggression with a further reduction in the second survey as staff become more confident in using zonal observations.

Background: PICUs often rely on enhanced observations, such as 1:1 or 2:1, to reduce violence and aggression. However, these practices have limited evidence of effectiveness and are frequently perceived negatively by staff and patients. At Willow Suite, a 12-bed male PICU, zonal observations were introduced in January 2024 as a less restrictive alternative. This approach involved designating staff to specific zones for proactive engagement with patients while maintaining safety and improving patient experience.

**Methods:** Data were collected from clinical records and incident reporting systems for three periods: pre-implementation (November–December 2023), immediate post-implementation (January–February 2024), and 10 months after implementation (November–December 2024). Key metrics included incidents of violence, seclusion episodes, and the duration of enhanced observations.

**Results:** The duration of enhanced observations reduced significantly, from a total of 51 days to 22 days in the first 2 months and maintained the same 10 months later. The average length of enhanced observations decreased by 58% immediately post-implementation, from 8.5 days per incident to 3.6, and further reduced to 3.1 days after 10 months. Seclusion episodes initially increased from 6 to 11 as staff were adapting to the new system, but the average length of seclusion dropped from 3.2 to 2.2 days with 55% of seclusions lasting a day or less. After 10 months, seclusion incidents had reduced further to 10 with average length of 2.5 days.

The length of all restrictions combined reduced from 70 days (average length 5.8 days) to 17 (average 2.7) in the first 2 months and to 11 (average 2.9) 10 months later.

There was no increase in incidents of violence and aggression in the initial 2 months and a reduction 10 months later.

**Conclusion:** The results suggest that zonal observations successfully maintained safety while reducing restrictive practices in our PICU over a one-year period. Other benefits observed were improvement in staffing consistency, increased staff confidence in managing clinical risks as well as patients reporting improvement in overall experience and engagement.

